# Correction: The impact of textual elements on the comprehensibility of drug label instructions (DLIs): A systematic review

**DOI:** 10.1371/journal.pone.0258020

**Published:** 2021-09-23

**Authors:** The PLOS ONE Staff

Figs 3 and 4 are incorrect. The publisher apologizes for the error. The authors have provided a corrected version here.

**Fig 3:**
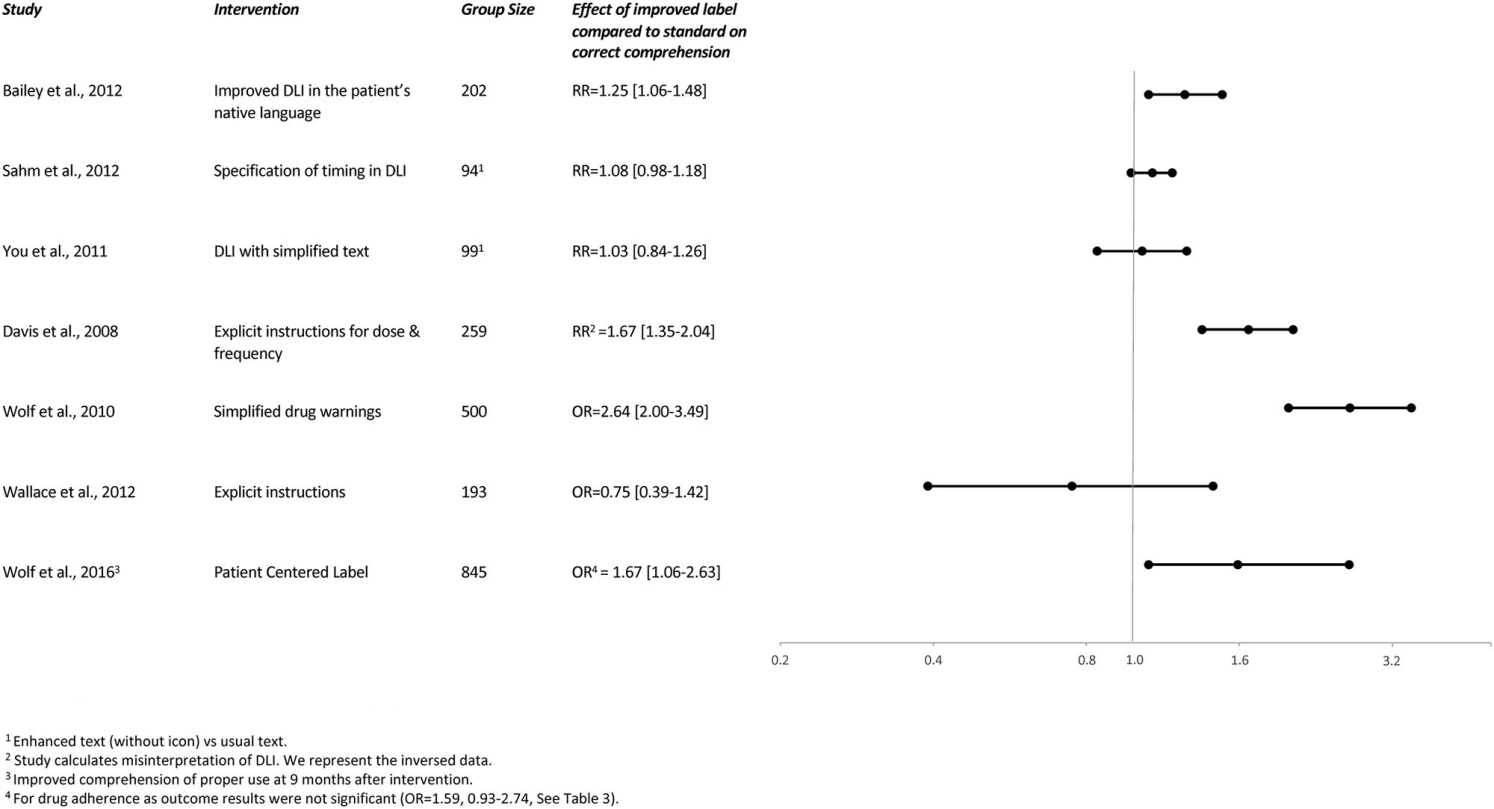
Relative risks and odds ratios of interventions in DLI on correct comprehension.

**Fig 4:**
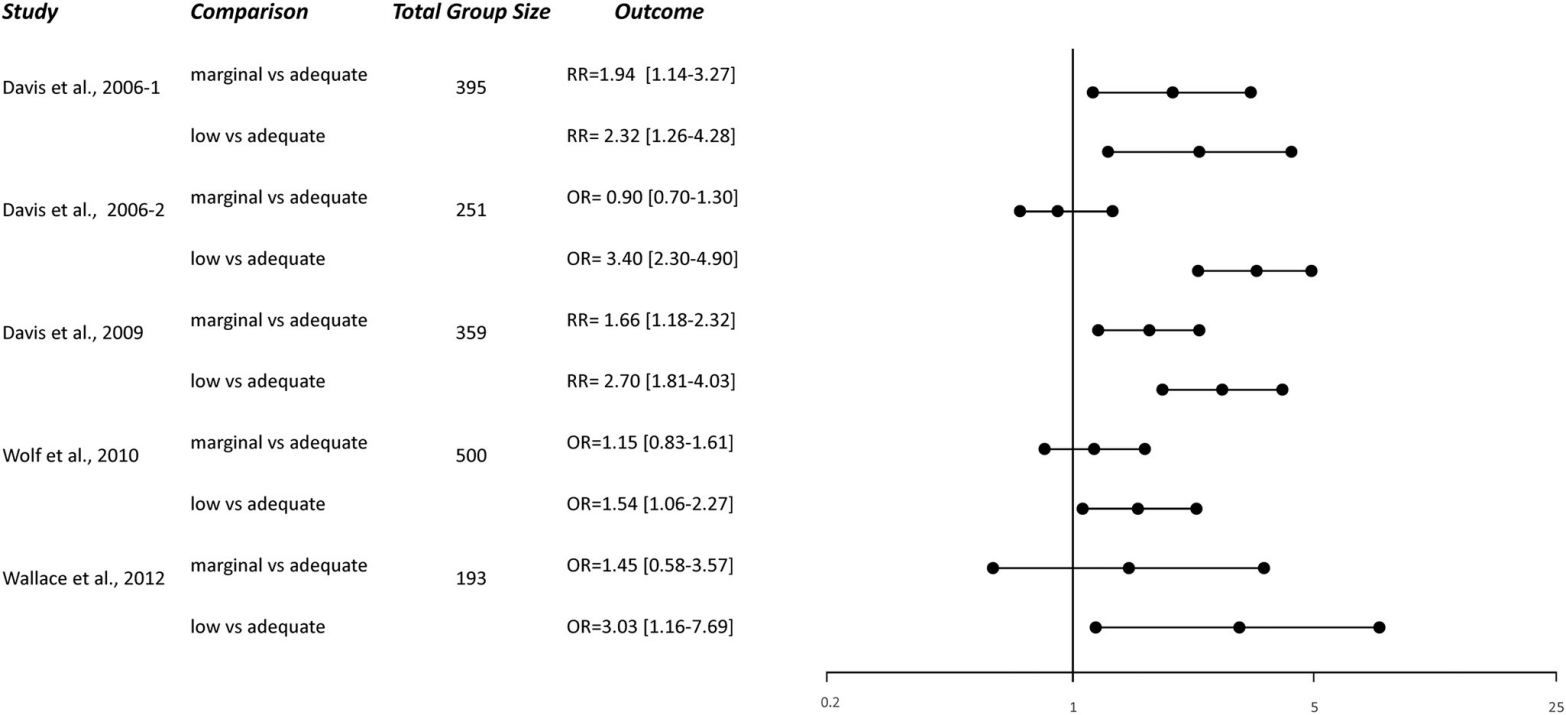
Relative risks and odds ratios of misunderstanding of patients with marginal/low health literacy compared to patients with adequate health literacy.
