# Supplementary material for: The impact of textual elements on the comprehensibility of drug label instructions (DLIs): A systematic review
Source: PLoS One. 2021 May 19;16(5):e0250238. doi: 10.1371/journal.pone.0250238 (PMC8133485; doi:10.1371/journal.pone.0250238)
Supplement: Attachment — Submitted filename: Response to reviewers.docx [file pone.0250238.s002.docx]

**Response to reviewers**

*Maghroudi et al.* The impact of textual elements on the comprehensibility of drug label instructions (DLIs): a systematic review.

We would like to thank the reviewer and the Editor for their valuable comments, and the possibilities to improve the paper considerably. As first major improvement, we have distinguished the concepts literacy and (functional) health literacy, and describe how these concepts were used in the individual studies. Second, we summarized the most important quantitative data in three figures that we added, describing (1) an overview of the effects of intervention studies between intervention and control groups, (2) an overview of differences found between people with adequate versus marginal/low health literacy, and (3) the quantitative findings concerning the textual elements that were investigated in the studies. However, given the heterogeneity of the results, the main part of this review remains a qualitative synthesis. We describe the process more clearly in our Methods section. The third major improvement we have made was rewriting the language and grammar of the paper after external proofreading. Added with the other comments, we believe we were able to improve the manuscript considerably, and are open to discuss further improvements.

| Reviewer #1: | |
| --- | --- |
| **Comments and/or suggestions** | **Author response** |
| 1. This manuscript is difficult to read owing to many atypical and/or erroneous lexical choices. In fact, it took so much effort to read the manuscript that I fear that I might have missed questions and details to help the authors improve the paper. I would recommend that, before resubmission to this or another journal, the authors ask someone who is not affiliated with the manuscript to act as a proofreader. | **Changed**  We thank the reviewer for this remark, and the specific suggestions to improve the paper. After the first revision by the first author, a proofreader has extensively commented on the manuscript, and we have rewritten the manuscript. |
| 2. Several of the studies deal with literacy (i.e., “reading level,” e.g., Davis et al., 2006) rather than “health literacy.” I believe that the authors should separate these two constructs, re-assign those studies, and rework all sections of the paper to include "health literacy" and "literacy" as different constructs. Not understanding health, healthcare, and treatment protocols is a very different problem from not having the reading skill to comprehend the meaning of “take two pills twice per day.” I suspect that this problem might have native language vs. language of label as a confound, as many people are differentially literate in different languages they speak, in which case the authors would need to consider that separately. | **Changed**  We agree with the reviewer that the constructs literacy and health literacy are different and need further clarification. First, we defined literacy and health literacy in our introduction, and explained the similarity between literacy and functional health literacy. Next, we explained that health literacy is a broader concept, yet most instruments measure only the functional component. In our results section, we analyse if the studies included in this review measured health literacy as functional health literacy, or as a broader construct. Finally, we reflect on this issue in our discussion.  **[line 85-97 in the Introduction; line 383-388 in the Discussion]** |
| 3. A smaller detail: there is a large literature demonstrating that patients forget or misremember as much as 75% of what healthcare professionals say during appointments; I’m surprised not to see a single citation for this. (Citations in paragraph 1, pp 4 refer to written instructions.) Many of these studies also include health-related stress variables which would strengthen the discussion on pp 17. | **Changed**  Thank you for this comment. Studies of Kessels (2003) and Linn et al. (2013) stated that 40-80% of the spoken information is forgotten, misremembered or remembered inaccurately. We added these studies in the introduction.  *“Between 40 to 80 per cent of the information during patient-physician encounters is forgotten, or remembered inaccurately [2-4] and information in patient leaflets is often considered as too complex [5-7].”* **[line 63-65]**  We would also like to thank you for the suggestion to include health-related stress variables. However, we choose to not include this specific variables, as we do not see the direct relation with the comprehension with the comprehensibility of DLIs. |
| (technical aspects of the study)  4. The inclusion of the PRISMA checklist and associated files makes it very clear that the method used for this study was systematic. I did have a difficult time understanding Table 2, however. I believe, based on the text on page 9 (15 of the pdf), that Table 2 refers to assessment of methodological quality based on an assessment from the Joanna Briggs Institute, and labels the questions used as Q1, Q2, Q3…Q13. It would be much easier for the reader who is not intimately familiar with this checklist if the topic areas of the questions were included on the table along with the question numbers. | **Changed**  We indeed used the critical assessment tools for methodological quality from the Joanna Briggs Institute, for the different study designs. As the questions among the different assessment checklists vary, we propose to attach the assessment checklists we used in Appendix 1, and refer to them in the Methods section.  In Table 2 we skipped the column inclusion (yes/no) – not marked with track changed – because the Table only mentions included studies as we state in the study title. |
| (technical aspects of the study)  5. On pp 8, the authors use text to describe some of the measures they took to maintain internal reliability and validity. There are some details here that I don’t understand: a. Why were 20% of the studies independently assessed by title and abstract, and then fully assessed? Were these different assessments? What was inter-assessor agreement during this process? If I understand correctly, after this process, the remaining 80% of the studies were assessed by only one person and no inter-assessor reliability was calculated. It would be good to know what the initial agreement was to understand why the bulk of the studies were assessed without further reliability measures. | **Changed:**  After minor differences between both assessors and 100% agreement after discussion, we decided to assess the remaining studies by only one person. In the text we mention Cohen’s Kappa for the inter-assessor reliability for the first 20%.  We have rewritten this paragraph as follows:  *Twenty per cent of the identified titles and abstracts were reviewed by the two independent reviewers (EM and HB). To validate the assessment, Cohen’s kappas were calculated and the inter-rater reliability was good (κ = 0.78).* **[line 137-140]** |
| 6. How did the authors determine that papers were “obvious duplicates?” Are these papers that were listed in multiple databases, or where the journal listing and the funding listing were retrieved separately? | **Changed**  What we did initially, was checking for duplicate studies in multiple databases. In the previous version, we did not describe duplicate publications of the same study results. In the revised version we did also mention these duplicates, we also explicitly mention two studies that published their results in 4 publications.  We described the process for duplicate studies more clearly in our Methods **[line 136-137; line 152-153]** and Results section, and we modified Figure 1 and changed Table 3 accordingly. Four publications (Wolf 2007 and Davis 2006-1, Shiyanbola 2016 and 2017) were based on the same study: those of Wolf 2007 and Davis 2006-1, just as the studies of Shiyanbola 2016 and 2017.  We believe it is more correct to speak of 28 studies that were presented in 30 publications. |
| 7. Did both EM and HvB complete the JBI assessment for all of the studies, or did one do only 30% as in the preceding “Data Classification and Analysis” section? | **Changed**  Both authors (EM and HvB) completed the Joanna Briggs assessment for all studies. The authors assessed the studies independently, and the inter-rater reliability was, after discussion, 100%. |
| (technical aspects of the study)  8. pp 14: What do the following stand for: REALM, NVS-d, SBSQ-d, SAHLSA, TOFHLA? | **Changed**  The acronyms stand for the health literacy measures that were used in the included studies. The acronyms for these health literacy measurements are written out when first mentioned in the manuscript **[line 99-103]**, and have been added as footnote under Table 3 **[line 231-232]**. |
| (technical aspects of the study)  9. I am accustomed to seeing meta-analyses that include effect sizes. Effect size analysis might not make statistical sense in this case, but I do think the paper would be stronger if there were a way to see graphically the group sizes and proportion of participant responses indicating concern for each item. E.g., for specificity of dosage instructions, what proportion of the participants in each listed articles reported that implicit wording was helpful. (I think this would involve including article 26 in this group). This would change table 4 considerably; perhaps Table 4 would become several figures. | **Changed**  We understand this suggestion, and we used this suggestion to graphically present the quantitative findings that were found in the studies. We took the opportunity to use this suggestion to look some broader than the specific suggestion of the reviewer to graphically compare the findings concerning the textual elements we found. We visualised the major quantitative data presented in the studies in new Figures ( 2, 3 and 4), we added quantitative data to Table 4, however we propose to replace Table 4 in the main by the figures 2-4, and move Table 4 to the Appendix.  Figure 2 presents the frequency of textual elements in the included studies.  Differences found between people with adequate versus marginal/low health literacy are presented in Figure 3, and Figure 4 presents the effect of textual intervention studies between intervention and control.  Finally in Table 4 (now Appendix 2), we added specific information with regard to the textual elements we found.    We changed the Methods section accordingly, and we discuss the findings in the Figures in the discussion section. |

| Editioral comments | |
| --- | --- |
| Thank you for submitting your manuscript to PLOS ONE. After careful consideration, we feel that it has merit but does not fully meet PLOS ONE’s publication criteria as it currently stands. Therefore, we invite you to submit a revised version of the manuscript that addresses the points raised during the review process.   We would appreciate receiving your revised manuscript by May 11 2020 11:59PM. When you are ready to submit your revision, log on to <https://www.editorialmanager.com/pone/> and select the 'Submissions Needing Revision' folder to locate your manuscript file. | **Changed**  In this document, we point-wise explain how we improved the paper with the comments of the Reviewer and the Editor. |
| Please include the following items when submitting your revised manuscript:   1. A rebuttal letter that responds to each point raised by the academic editor and reviewer(s). This letter should be uploaded as separate file and labeled 'Response to Reviewers'. 2. A marked-up copy of your manuscript that highlights changes made to the original version. This file should be uploaded as separate file and labeled 'Revised Manuscript with Track Changes'. 3. An unmarked version of your revised paper without tracked changes. This file should be uploaded as separate file and labeled 'Manuscript' | **Changed**  All items are submitted in the Editorial Manager Submission System. |
| 4. Please include full names and then acronyms so that readers do not become confused. | **Changed**  The acronyms stand for the health literacy measures that were used in the included studies. The acronyms for these health literacy measurements are written out when first mentioned in the manuscript, and added as footnote under Table 4. |
| 5. With respect to effect sizes, there are a number of traditional and nontraditional ways to calculate effect sizes ranging from Cohen’s d to Standard Mean Difference (SMD), so I would encourage the authors to consult with a statistician to determine if there are ways to represent the findings in a way to evaluate participant responses in a meaningful way. | **Changed**  We would also thank you for this consideration, see also our reply to comment 4 of reviewer #1. We explored possibilities to summarize effect sizes, and we believe a graphical presentation of the findings is the best way to visualize the effect size found in the different studies. We checked this approach with statisticians, and they mentioned that this approach was more meaningful than calculation of mean effect sizes, given the heterogeneity of the data. We mention this point in the limitations section of our discussion. |

| Journal requirements | |
| --- | --- |
| **Comments and/or suggestions** | **Author response** |
| Please ensure that your manuscript meets PLOS ONE's style requirements, including those for file naming. The PLOS ONE style templates can be found at <http://www.plosone.org/attachments/PLOSOne_formatting_sample_main_body.pdf>  and <http://www.plosone.org/attachments/PLOSOne_formatting_sample_title_authors_affiliations.pdf> | We believe our manuscript meets PLOS ONE’s requirements. |
| Please include your tables as part of your main manuscript and remove the individual files. Please note that supplementary tables (should remain/ be uploaded) as separate "supporting information" files | **Changed**  Tables are included in the manuscript directly after the paragraph in which they are first cited. |
| PLOS requires an ORCID iD for the corresponding author in Editorial Manager on papers submitted after December 6th, 2016. Please ensure that you have an ORCID iD and that it is validated in Editorial Manager. To do this, go to ‘Update my Information’ (in the upper left-hand corner of the main menu), and click on the Fetch/Validate link next to the ORCID field. This will take you to the ORCID site and allow you to create a new iD or authenticate a pre-existing iD in Editorial Manager. Please see the following video for instructions on linking an ORCID iD to your Editorial Manager account: <https://www.youtube.com/watch?v=_xcclfuvtxQ> |  |
| We note you have included a table to which you do not refer in the text of your manuscript. Please ensure that you refer to Table 3 in your text; if accepted, production will need this reference to link the reader to the Table. | **Changed**  Table 3 is included and referred to in the manuscript. |
| Thank you for stating the following in the Funding details of your manuscript:  "This work was supported by ZonMw, under Grant 848022004."  We note that you have provided funding information that is not currently declared in your Funding Statement. However, funding information should not appear in the Acknowledgments section or other areas of your manuscript. We will only publish funding information present in the Funding Statement section of the online submission form. Please remove any funding-related text from the manuscript and let us know how you would like to update your Funding Statement. Currently, your Funding Statement reads as follows: "The funders had no role in study design, data collection and analysis, decision to publish, or preparation of the manuscript. " | **Changed**  We removed the funding information from the Acknowledgments section and we will declare this information in our Funding Statement.  Our Funding Statement will be described as: “*This work was supported by ZonMw, under Grant 848022004.”* |
