# Supplementary material for: The impact of textual elements on the comprehensibility of drug label instructions (DLIs): A systematic review
Source: PLoS One. 2021 May 19;16(5):e0250238. doi: 10.1371/journal.pone.0250238 (PMC8133485; doi:10.1371/journal.pone.0250238)
Supplement: Attachment — Submitted filename: Response to reviewers.docx [file pone.0250238.s003.docx]

Response to reviewers

Maghroudi et al. The impact of textual elements on the comprehensibility of drug label instructions (DLIs): a systematic review.

We would like to thank the Reviewers and the Editor for their valuable comments and suggestions, and the possibilities to improve the paper considerably. In our response we explain point-wise how we improved the paper. We would like to mention the 5 major improvements:

(1) The second author (CvH) has extensively copy-edited the manuscript. This period, the first (and corresponding) author had little time available to take the lead in this extensive revision as she was responsible for intensive (online) education for nurses about health communication. CvH lead and drafted the revision. We believe that a shared first authorship is the best way to value the contributions of EM and CvH.
(2) We have explained in more detail what is meant by ‘comprehension of DLIs’ throughout the manuscript.
(3) We explain in the Introduction and Discussion what is measured by different health literacy instruments and how this is related to the comprehension of DLIs.
(4) All recommendations on the use of textual elements are accompanied with examples in Table 3 and in the Results section.
(5) In the Discussion section we synthesize the key findings of our review and only address the interpretation of the data presented.

Added with the other comments, we believe we addressed all issues, and are open to discuss further improvements.

| Reviewer #2: | |
| --- | --- |
| Comments and/or suggestions | Author response |
| 1. The manuscript would benefit from additional copyediting for typographical and grammatical issues. | **Changed.**  The second author has extensively copy-edited the manuscript. |
| 2. At various points, I found the manuscript difficult to follow and the synthesis of the findings lacking. While it seems that much care was taken to conduct a careful review and summary of the papers, the resulting synthesis and conclusions are less compelling.   For example, the Wording section on pgs. 24-25 states that complexity is shown to be problematic; however, it’s not clear what specific ways to simplify would be helpful. In other words, complexity is stated to be an issue, but it’s not clear what a solution or effective simplification interventions have been shown to help.  Similarly, the Dose Measurements section on pg. 25 doesn’t indicate alternative words that would be preferable to those discouraged. Do these prior studies not offer any substantive recommendations on how to improve thing | **Changed.**  We changed the Wording section in the synthesis of the results. This section is now referred to as Plain language [lines 310-317].  We explain medical jargon and complex words should be avoided and which simplifications might be a solution. We also mention these simplifications in Table 3 in the column ‘Recommendations concerning textual elements’.  Unfortunately, prior studies do not offer substantive recommendations on alternative words that can be used to indicate dose measurements. We have explicated this in the dose measurements sections [lines 321-327]. See also Table 3, column ‘Recommendations concerning textual elements’. |
| 3. Another concern is that the Discussion section sometimes diverges from the actual findings into relatively speculative claims (e.g., suggesting that meal intervals not be used). While the authors acknowledge this has not been investigated specifically, it doesn’t necessarily seem to follow from the findings of this review. Further, the Implications for Research and Implications for Practice sections also seem to contain content that is only tangentially related to these topics and disconnected from the findings. | **Changed.**  In the discussion section, the claims about avoiding meal intervals in dosage instructions have been removed [lines 400-408]. Moreover, the sections ‘Implications for research’ [lines 479-487] and ‘Implications for practice’ [lines 489-495] are now in line with the findings from the literature review. |
| 4. While limiting the scope of the research is necessary due to practical limitations, excluding work on visual elements of labels may be problematic. Indeed, there is a substantial amount of work examining how visual elements (e.g., icons) can be used to improve comprehension and other outcomes among lower (health) literacy groups. This could be one reason the review did not find many studies specifically examining textual interventions aimed at improving outcomes among individuals of lower literacy. In other words, because of the challenges that low literacy presents to processing textual information, researchers have likely looked elsewhere to graphics, icons, or other visual elements as solutions. | **Changed.**  We agree with the reviewer that patients with limited health literacy experience problems with processing textual information, and presenting information in graphics, icons, or other visual elements might be a solution. However, research from Sletvold et al. (2019) and Katz et al (2006) show visual elements should be combined with textual information in order to facilitate patients’ comprehension of medical instructions. We addressed the limitation of excluding work on visual elements on labels in the Discussion section [lines 452-460]. Moreover, in the Introduction section we acknowledge that substantial work has been conducted on using visual elements to improve patients’ comprehension, but mixed results have been found regarding their effectiveness [lines 127-136]. |
| 5. With so much of the paper focused on health literacy, it would also be helpful to explain the specific measures in more detail. For example, the REALM essentially measures an individual’s familiarity with a list of various medical terms and thus seems inherently linked to someone’s ability to interpret more complex terms on a medication label. How do the other measures compare to this? | **Changed.**  In the Introduction we have added more information about the specific measures of health literacy instruments [lines 104-120]. Also, in the discussion section we reflect on health literacy measures and how this relates to patients’ comprehension of DLIs (i.e., rephrasing the instruction in your own words vs. demonstrating proper drug use) [lines 424-433]. |
| 6. It appears that the vast majority of studies mentioned focus on medications that are dispensed via a health care professional (e.g., pharmacist or doctor). Are studies of drugs that can be accessed without an intermediary (e.g., over-the-counter drugs in the United States) also included? | **Changed.**  In Table 3, we mention the type of medications (prescribed vs. over-the-counter drugs) used in the studies. Also, the Results section includes information about the number of studies focusing on DLIs of prescribed drugs, over-the-counter drugs, or both [lines 255-257]. |

| Reviewer #3: | |
| --- | --- |
| Comments and/or suggestions | Author response |
| 1. The authors don't define "comprehension" in the manuscript, but it is important to do so. The studies included in the review will have measured "comprehension" in different ways. For example, some studies will ask participants to verbally describe what they will do, others will require participants to demonstrate (in some manner) what they will do (i.e. to demonstrate how many tablets they would take and when) and others will require both. This should be clarified throughout | **Changed.**  We agree with the reviewer that we should have defined ‘comprehension’. Throughout the manuscript we have explained what is meant by patient’s comprehension of DLIs.   - Introduction: lines 85-87; - Table 3: column primary outcome; - Results: lines 257-263; - Discussion: lines 424-433. |
| 2. Like the editorial comments, I think there are ways the authors could estimate effect sizes despite the different approaches and measures used in the studies. It is not clear that the authors are referring to heterogeneity in the statistical sense when offering that as a reason against combining studies with quantitative measures. At minimum, the way the current figures showing RR and OR for various groups needs to be more clearly described and defended. | **Changed.**  In the context of the heterogeneity in interventions, setting and study population, we believe it is not correct to pool outcomes. We agree with the earlier suggestions by the reviewers and Editor that graphical illustration of the effect of the interventions and health literacy on comprehension is valuable. In the current version, we motivate our approach in the Methods section, including choice for showing OR/RR and their confidence intervals. [lines: 218-230] |
| 3. More examples of explicit vs. 'implicit' dose instructions and "wording of dose measurements" is needed. | **Changed.**  We have added examples of dose instructions in Table 3 (columns: ‘Findings of textual elements (TE) and health literacy (HL)’, ‘Recommendations concerning textual elements’) as well as in the Results section: lines 296-308.  We have added examples of dose measurements in Table 3 (columns: ‘Findings of textual elements (TE) and health literacy (HL)’, ‘Recommendations concerning textual elements’) as well as in the Results section: lines 321- 327. |
| 4. The statements provided in the results section are often not sufficiently specific  - Line 260 "DLIs with explicit instructions are effective" at what? - Line 264 information about "wording" is not sufficiently clear. Examples would help - Line 281 placing each part of the instruction on a separate line was "proposed as an effective way": is there data to support this? - Line 288 presentation of numbers is "preferred": the review is focused on 'comprehension' is there data on comprehension? - Line 299 native language is "recommended": is there data to support this - Line 318: "a negative relation between comprehension of DLI and health literacy" suggest a **negative correlation**, but this is not what you have found. - Line 318 the information on health literacy should better recognise that many studies have found benefits of textual components of DLI improving comprehension for people with both low and high health literacy (though a larger effect in people with low health literacy) | **Changed.**  The section about the specificity of dosage instructions has been revised. The sentence in line 260 has been removed.  The section ‘wording’ has been revised and now referred to as ‘plain language’ [lines 310 – 317]. Examples of medical jargon and difficult words are provided as well as their simplifications.  The section ‘number of messages per instruction line’ has been revised. In this section we refer to the studies who have proposed to place each part of the instruction on a separate line.  The section ‘presentation of numbers’ has been revised and we only address studies in which the comprehension of numbers in DLIs is investigated.  Data that support this recommendation are presented in Table 3 (studies of Alburikan 2018, Bailey 2012, Koster 2014).  The specific sentence in line 318 has been removed  The section on health literacy and drug label understanding has been revised. We have explained the results about the relation on patients’ comprehension of DLIs and health literacy in more detail [lines 364-388]. |
| 5. The lack of specificity in the results makes it hard to say that the key findings as presented in the first paragraph in the Discussion have been demonstrated. | **Changed.**  We present our key findings in the first paragraph of the Discussion section [lines 390-399]. |
| 6. Line 356 it is not clear what the authors are including in "plain language". | **Changed.**  In the Results section [line 310-317] as well as in the Discussion section [line 408-414] we explain what we included as ‘plain language’. |
| 7. Line 358 you don't appear to have provided clear evidence regarding the use of "dayparts" as opposed to alternative ways to specify the dose. Please clarify. | **Changed.**  In the Discussion section the speculative claims about avoiding meal intervals in dosage instructions have been removed [lines 404-414]. Moreover, the sections ‘Implications for research’ [lines 479-487] and ‘Implications for practice’ [lines 489-495] are in line with the findings from the literature review. |

| Editioral comments | |
| --- | --- |
| 1. While the writing is much better in this revision, there remain a number of organizational, typographical, spelling, grammatical and syntactic errors related to the use of English; as well as a number of issues with vague and ambiguous descriptions of terms, outcome measures, and effect sizes. These issues contribute to difficulty in understanding the results and the interpretation of the results in the discussion section. Both reviewers highlight these issues in their comments. | **Changed.**  The second author has extensively copy-edited the manuscript.  We believe a shared first authorship is the best way to value the contributions of CvH. |
| 2. The discussion section often departs to topics and/or conclusions that are not directly supported in the data. The reviewers noted this in their comments and I agree that refocusing the discussion to only address the interpretation of the data that is presented would greatly improve the paper. | **Changed.**  In the Discussion section the speculative claims about avoiding meal intervals in dosage instructions have been removed [lines 404-414]. |
