# Supplementary material for: The impact of textual elements on the comprehensibility of drug label instructions (DLIs): A systematic review
Source: PLoS One. 2021 May 19;16(5):e0250238. doi: 10.1371/journal.pone.0250238 (PMC8133485; doi:10.1371/journal.pone.0250238)
Supplement: Attachment — Submitted filename: Response to reviewers_revisie3_final.docx [file pone.0250238.s004.docx]

Response to reviewers

Maghroudi et al. The impact of textual elements on the comprehensibility of drug label instructions (DLIs): a systematic review.

We would like to thank the Reviewers and the Editor for their valuable comments and suggestions, and the possibilities to improve the paper. In our response we explain point-wise how we improved the paper. We would like to mention the 4 minor improvements:

(1) Adjustments to the manuscript have been made based on the minor textual suggestions of Reviewer #2.
(2) We clarify the statement about how the two reviewers decided to exclude studies based on the titles and abstract. To determine the reliability of this decision, twenty per cent of the sample was double coded by the reviewers, after which the Cohen’s kappa was computed.
(3) We explain in the Discussion what is meant by our recommendation about implementing optimized DLI’s in health information systems.
(4) The aim of the paper is emphasized in the Abstract. Also, a research question is added in the Introduction.

Added with the other comments, we believe we addressed all issues.

| Reviewer #2: | |
| --- | --- |
| Comments and/or suggestions | Author response |
| 1. Textual suggestions:  - Line 48 – Consider changing “whom” to “which”. - Line 325 – replace “did no” with “did not”. - Line 388 – nine months. - Line 406 – “to specify the” instead of “to specify to”. - Line 464 – should it be drug “regimen” instead of “regime”? | Changed. |
| 2. Line 160-161 - Might add some text to clarify the following statement: “Twenty per cent of the identified titles and abstracts were reviewed by the two independent reviewers (EM and HB).” Does this indicate a sample was drawn to make the workload more manageable? | Changed.  We clarified the statement about how the two reviewers decided to exclude studies based on the titles and abstract. To determine the reliability of this decision, twenty per cent of the sample was double coded by the reviewers, after which the Cohen’s kappa was computed. [lines 202-206]. |
| 3. Line 242 – summarize inclusion criteria or remind the reader of where this was discussed previously. | Changed.  We summarized the inclusion criteria [lines 291- 292]. |
| 4. Lines 492-494 – I didn’t understand the following recommendation: “Depending on the health care context, DLIs can be optimized by adapting the associated codes in these information systems.” | Changed.  We explained this recommendation in more detail in the Discussion section [lines 544-552]. |
| 5. Table 2 – Consider adding a footnote under table 2 that lists the JBI checklist questions. | Changed.  We considered listing the JBI checklist questions as a footnote under table 2. However, different JBI checklists have been used (i.e., cross-sectional studies, experimental studies, qualitative studies, and randomized controlled trials), which would result a big list of questions. We therefore added a footnote in which we refer to the online supplementary files for the specific questions per JBI checklist. |
| 6. Figure 3: Should the vertical line in the figure be at 1.0 on the x-axis? | Changed.  We changed the Figure 3: the vertical line is at the 1.0 on the x-axis. We also noticed (and corrected) the use of comma’s instead of points in the numbers under the x-axis. |

| Reviewer #4: | |
| --- | --- |
| Comments and/or suggestions | Author response |
| 1. Although the methods seemed reasonable, I was not sure what the actual research question or purpose was. There is already a lot done in this area, and there were several references with studies on the relationship between drug label info and FHL that were not even referenced. That makes the whole search strategy curious. The implications for creating a new label with some key points has been in existence since almost a decade so I am unsure what new knowledge has been added here. | Changed.  The aim of the paper is emphasized in the Abstract [lines: 41-. Also, a research question is added in the Introduction [lines 152-156].  As regard to what this study adds: though other studies have been done concerning the comprehensibility of DLI’s, this is -to our knowledge- the first review that systematically investigates the concrete textual elements that facilitate or hinder correct interpretation of DLI's in relation to patients' health literacy [Discussion: lines 479 – 484].  We thank the reviewer for her/his remark concerning the search strategy. We erroneously mentioned health literacy as element of the search strategy in the text in the search strategy paragraph. Health Literacy is no element of the search strategy (see Table 1), but is an important variable in the data extraction and analysis (see data extraction and analysis). We removed health literacy from the search strategy paragraph, and summarized the correct inclusion criteria in the Results section (selection of studies, see also comment 3, reviewer #2). |
